# Bridge Plate Failure with Extensor Tendon Injury: A Case Report and Literature Review

**DOI:** 10.1155/2018/3256891

**Published:** 2018-10-29

**Authors:** Rachel Lefebvre, Jessica Intravia, Lisa Cao, Alidad Ghiassi, Milan Stevanovic

**Affiliations:** Division of Hand Surgery, Department of Orthopaedic Surgery, University of Southern California, 1200 N State Street CT-A7D, Los Angeles 90033, USA

## Abstract

**Background:**

Dorsal spanning plate fixation is an increasingly popular method of fixation for certain distal radius fractures. Published outcomes are encouraging, but complications are still reported.

**Methods:**

We present a case report of a 26-year-old woman with spanning plate breakage and extensor pollicis longus (EPL) metallosis, fraying, and near-complete rupture. The patient's unsuccessful follow-up led to this complication. Metallosis and damage to the extensor indices (EI) and distal extensor carpi radialis longus (ECRL) were intraoperative obstacles for tendon transfer to restore EPL function.

**Results:**

Tendon transfer in the setting of bridge plate failure has not yet been described in the literature. There are reports of spanning plate failure indicating that breakage often happens through the plate's holes and after fracture healing.

**Conclusions:**

The compounded complication of plate failure with extensor tendon injury emphasizes the important relationship between the local anatomy, barriers to patient care, and potential problems after spanning plate fixation.

## 1. Introduction

Tendon injury and hardware failure after dorsal spanning plate fixation are not common, but have been reported as independent complications [1, 2]. We present a case of extensor tendon damage including extensor pollicis longus (EPL) near-rupture in the setting of a retained, broken dorsal bridge plate. Intraoperative findings suggest that hardware breakage led to tendon injury. We review the patient's presentation, intraoperative findings, and the literature about complications of dorsal spanning plate fixation.

## 2. Case Report

**Figure 1:**
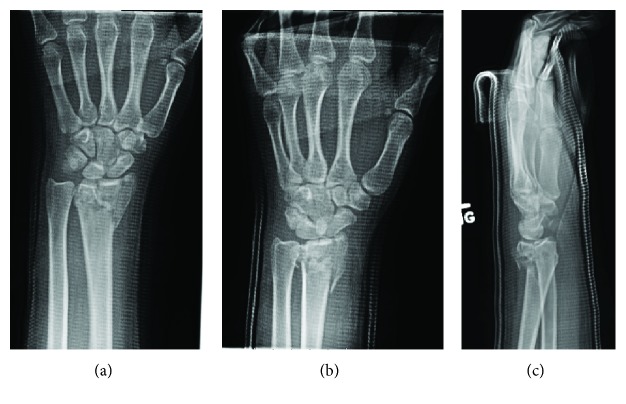
Radiographic presentation. Initial imaging on presentation to hand clinic after the patient was splinted at another facility.

**Figure 2:**
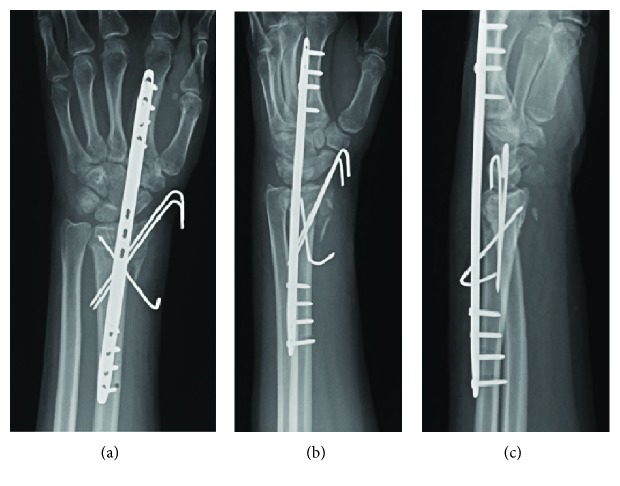
Initial fixation. Postoperative X-ray showing fixation with a 2.4/2.7 mm Synthes dorsal spanning plate and three K wires.

A 25-year-old, right-hand-dominant woman presented to clinic nine days after she fell from a bunk bed. The patient was otherwise healthy, but had a history of intravenous drug abuse and incarceration. X-rays of the left wrist were taken through a splint applied the day before at another facility (Figure 1). Since this fracture was highly comminuted with small, intra-articular fragments, we elected to restore overall radiographic parameters and relative stability using ligamentotaxis with a 2.4/2.7 mm Synthes dorsal bridge plate. Additional, percutaneously placed K wires offered additional stabilization of the radial styloid and lunate facet (Figure 2). To allow staged hardware removal without multiple trips to the operating room, K wire ends were left outside the skin. The bridge plate was placed with two incisions—one over the index metacarpal and one over the radial shaft. The third dorsal extensor compartment at the level of the wrist was not opened, in contrast to descriptions of others' technique [3]. However, the plate was visualized deep to extensor tendons in the proximal, radial shaft incision. Full passive finger motion was confirmed after plate placement indicating that the wrist had not been overdistracted. Postoperative X-rays showed improved radial inclination and articular congruity, but residual slight dorsal tilt (Figure 2).

The patient returned regularly for her initial follow-up visits; the two K wires were removed in clinic 4 weeks after surgery. At 4 weeks postoperatively, she was able to extend her thumb at the interphalangeal joint. Plate removal was discussed with the patient, but unfortunately, she was lost to follow-up and did not return for the next twelve and a half months.

**Figure 3:**
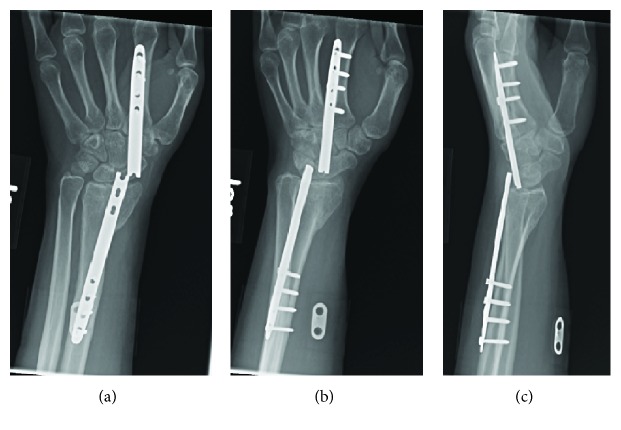
Return to clinic. X-rays on return to clinic, 12.5 months after dorsal bridge plate placement.

When she returned to clinic more than a year after surgery, she felt a clicking sensation with wrist motion. She estimated that this began three months ago. She was unable to extend her thumb for the past month. She denied any new trauma to the wrist or hand. The plate edges were prominent on physical exam, particularly with wrist flexion. She was unable to extend the distal phalanx of her thumb. X-rays showed that the dorsal bridge plate had broken at the level of the radiocarpal joint, through an empty screw hole (Figure 3). We recommended operative removal of hardware and EPL reconstruction. She did not have a palmaris longus (PL) on physical exam.

**Figure 4:**
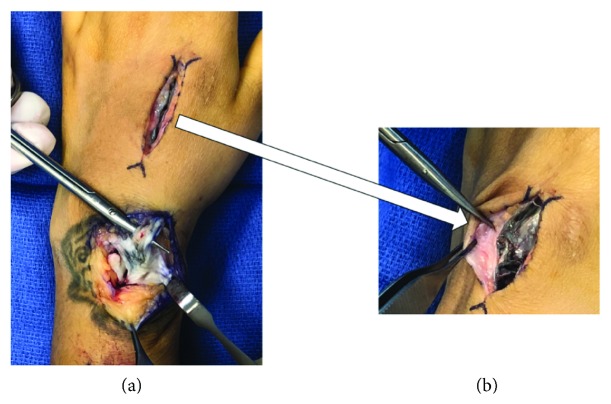
Extensor tendon damage. Metallosis, fraying, and near-complete rupture of the EPL tendon, shown on top of the tenotomy scissors (a). Close-up view of the metallosis as well as the distal EI and ECRL tendon damage over the index metacarpal (b).

**Figure 5:**
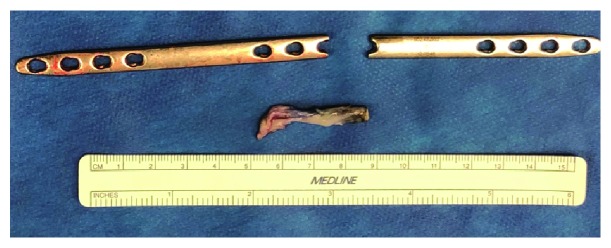
Damaged EPL and bridge plate. The excised portion of the frayed EPL tendon is photographed next to the broken dorsal spanning plate. This damaged portion of the EPL tendon was at the level of the broken plate in situ.

**Figure 6:**
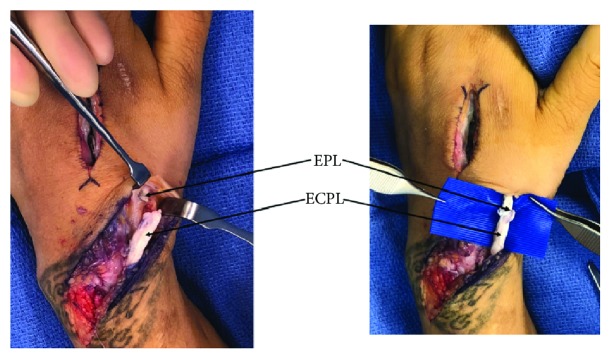
EPL reconstruction. We reconstructed the EPL tendon using an end-to-end tendon transfer from the ECRL.

In the operating room, we exposed the dorsal bridge plate through the two incisions used for plate placement. We made a third incision over the EPL at the level of the distal radius. We visualized the bridge plate deep to the extensor tendons in all incisions. Metallosis, severe attenuation, fraying, and a near-complete rupture of the EPL tendon was found at the same level the plate was broken (Figure 4(a)). We excised the damaged portion of the EPL tendon (Figure 5). We saw extensive metallosis and damaged-appearing extensor indices (EI) and distal extensor carpi radialis longus (ECRL) tendons over the index metacarpal (Figure 4(b)). We transferred ECRL to the EPL in an end-to-end fashion after resecting the damaged, distal portion of the ECRL tendon (Figure 6).

## 3. Discussion

Dorsal spanning plate fatigue is reported in the literature, but it is not common. In one series of 62 patients, a single patient's bridge plate broke [2]. In a larger study of 144 distal radius fractures treated with dorsal bridge plates, five cases of hardware breakage were reported [1]. In three of those five broken plates, the site of plate fatigue was through an empty screw hole at the level of the radiocarpal joint [1]. Authors have also postulated that a thicker plate of 3.5 mm may decrease the risk of plate fatigue [2]. In our patient, the 2.4/2.7 mm plate broke through a screw hole. In addition to considering using a thicker plate, we propose that future designs of the dorsal bridge plate could offer options without central holes. While the plate would still experience stress with attempted wrist motion, an updated design might decrease an inherent mechanical weakness of the current commercially available designs.

There may be a correlation between the length of time a plate is retained and the risk of plate breakage. In the study reporting one plate fracture, the patient's plate was in situ for 16 months before it failed [2]. In the series of five hardware fractures, plates broke between five and 20 months after placement, with an average failure time of 10 months [1]. In all five of those cases, fracture healing occurred before plate breakage [1]. From our patient's history, we believe the dorsal spanning plate broke around nine months after fracture fixation. Similar to descriptions in the literature, the distal radius fracture was healed and there was broken hardware on return presentation. Timely plate removal restores wrist motion and decreases the number of cycles that the joint-spanning implant is stressed. At our busy, urban, level one trauma center, we have recently begun scheduling plate removal at the time of insertion. This not only guides patients' expectations about their upcoming hardware removal, but also anecdotally improves follow-up.

Location of distal plate fixation is yet another consideration in minimizing the risk of bridge plate fatigue. While soft tissue injury and fracture pattern influence the decision regarding distal plate placement, studies have shown that distal fixation to the long finger metacarpal is biomechanically stronger [4]. Our patient's bridge plate was placed on the index metacarpal because this assisted with radiocarpal ulnar deviation and restoration of radial inclination and height. There is also evidence that placement on the index metacarpal versus the long finger metacarpal poses a lower risk for tendon entrapment [5]. However, it is important to consider that our choice of index metacarpal location for distal fixation may have placed our patient at increased risk for plate fatigue.

The second complication in our case is extensor tendon injury. There are reports of extensor tendon rupture with dorsal spanning fixation of distal radius fractures. In one case, rupture of the ECRL tendon occurred with plate removal [2]. In another case, fracture callus contributed to rupture of an EPL tendon [1]. Extensor tendon ruptures, particularly of the EPL, have more commonly been reported with nonoperative treatment or palmar fixation of distal radius fractures [6, 7]. Extensor tendon injury can be spontaneous, or it can be a result of trauma, fracture callus, or hardware [7–10]. There has not yet been a report in the literature of immediate tendon transfer for tendon rupture in the setting of a broken bridge plate.

Cadaveric studies support the suspected relationship between our patient's hardware failure and EPL injury. Extensor tendon entrapment during plate placement is possible [5, 11]. However, this did not seem to be a contributing factor in our case. Even when tendons are not entrapped, contact between tendons and hardware occurs. A study of dorsal spanning plates in 12 fresh frozen upper extremities fixed both to the index and long finger metacarpals showed that all plates were directly touching EPL tendons [11]. Contact between distal radius fixation and tendons has a recognized role in both flexor and extensor tendon injury [12–14]. Although less well described in the context of spanning fixation, it follows that friction between a spanning plate and extensor tendons could contribute to tendon damage. Furthermore, with hardware breakage, the smooth edges of a spanning plate become sharp and irritating to nearby tendons. In our case, metallosis and fraying of the EPL tendon at the same level as hardware fatigue gives compelling evidence that the two complications were related.

In our patient, additional metallosis and tendon damage were seen over the index metacarpal. This meant that the distal portions of the EI and ECRL tendons were not good candidates for tendon transfer to restore EPL function. We resected the distal, damaged portion of the ECRL and transferred the proximal, normal-appearing portion of the tendon to the EPL in an end-to-end fashion. Other tendon transfers, for example, from palmaris longus (if present) or an intercalary tendon graft are alternative motion-preserving surgical options. There is no published literature examining the degree of metallosis and potential EI and ECRL tendon damage in patients with retained spanning plates. In our clinical experience, there can be metallosis over the metacarpal, particularly if removal is delayed.

Routine removal of dorsal spanning fixation is recommended after plate placement [2, 15, 16]. In our institution, we remove dorsal bridge plates after fracture consolidation. Thorough education and access to care is necessary for patients to have timely removal of bridge plates. This can be especially challenging in trauma patients with severe wrist injuries in the setting of complex social situations. We caution careful use of spanning internal wrist fixation in patients with psychosocial issues or anticipated difficulty with follow-up. Our case suggests that neglected dorsal spanning plate failure may directly contribute to further tendon complications. In patients who have retained hardware and plate failure, we recommend immediate hardware removal to prevent or halt possible extensor tendon damage.
